# Differentially expressed microRNAs in aneuploid preimplantation blastocysts: a systematic review

**DOI:** 10.3389/frph.2024.1370341

**Published:** 2024-03-14

**Authors:** Arwa Almutlaq, Xavier Viñals Gonzalez, Sioban SenGupta

**Affiliations:** ^1^ Reproductive Health, Institute for Women’s Health, University College London (UCL), London, United Kingdom; ^2^ Clinical Laboratory Sciences, College of Applied Medical Sciences, King Saud University, Riyadh, Saudi Arabia

**Keywords:** microRNA (miRNA), aneuploidy, blastocyst, embryo quality, biomarker

## Abstract

**Introduction:**

MicroRNAs are small noncoding genes with gene expression regulatory function. Their emergence as potential diagnostic biomarker for many diseases has gained a specific interest among researchers. Observations of changes in miRNA levels correlating with aneuploidy in early embryos raise the prospective of employing miRNA as biomarkers to assess the embryo quality.

**Method:**

To identify and gather the miRNAs with potential link to chromosomal abnormalities in embryos from previous research, we conducted a systematic search using four databases, including Embase, Medline, Web of Science, and Cochrane databases in accordance with PRISMA guidelines.

**Results:**

Out of 200 identified records, only seven met the inclusion criteria. Seven miRNAs: miR-19b, miR-517c, miR-518e, miR-522, miR-92a, and miR-106a exhibited persistent downregulation in aneuploid blastocysts in the included studies. These miRNAs are members of important miRNA clusters, associated with abnormal expression in studies on reproductive failure. Pathway analysis revealed their involvement in regulating gene transcription, as well as cell cycle progression and apoptosis.

**Discussion:**

The changes detected in the miRNA expression in aneuploid embryos across different studies support the aneuploidy and miRNA relationship and prospect miRNA as a valuable tool for the assessment of embryo quality. Collectively, these observations highlight the role of miRNAs in embryonic development, and their involvement in genetic abnormalities that occur in embryos, such as aneuploidy, indicating their potential implementation to improve the embryo selection and reproductive outcomes.

## 1 Introduction

Chromosomal abnormalities are major factors that contribute to infertility, impacting over 50% of preimplantation embryos, and leading to reproductive failures or congenital defects. While morphological assessments have advanced embryo evaluation, preimplantation genetic testing has emerged as an additional diagnostic tool to enhance pregnancy odds by selecting aneuploidy-free embryos for transfer (1, 2). In the current preimplantation genetic testing (PGT) practice, the DNA is isolated from biopsied trophectoderm cells (TE) to identify chromosomal imbalances if present. Ultimately, the selection of embryos to transfer is influenced by the genetic testing for aneuploidy results. While selecting embryos without chromosomal aberrations has generally reduced the miscarriage rates, some euploid-diagnosed embryos fail to reach live births (3). Understanding the genetic basis of chromosomal abnormalities is important for a more indicative assessment of the embryo quality.

It has been previously suggested that blastocysts RNAs, particularly miRNAs, may provide informative insights into the developmental competence of embryo (4, 5). MiRNAs are a group of non-coding genes with 20–22 nucleotides, which gained popularity in clinical medicine as diagnostic markers for many diseases (6, 7). The role of miRNA in early development has been previously investigated, showing significant contribution in the process of implantation and stem cell differentiation (8–10). However, only limited studies have explored the potential of miRNAs in assessing developmental competence of early embryos, and the possible relationship between aneuploidy and miRNA expression (11–18). In this systematic review, our aim is to identify and combine the reported information on miRNAs with potential association to aneuploidy in preimplantation blastocysts.

## 2 Materials and methods

### 2.1 Search strategy

A systematic search was conducted across four databases, namely Embase and Medline (via Ovid), Web of Science and Cochrane clinical trials. Additional sources, including the UK National Research Register and the British Library (EThOS), were also searched. The search query used a set of keywords detailed in Table 1. Boolean operators (AND/OR) and parentheses were used to combine the keywords and refine the search results. No restrictions were applied regarding time or species, but only English-language transcripts were reviewed (Table 1). Only original sources, such as peer-reviewed journal articles, conference abstracts, theses, and dissertations, were included to ensure reliability of the results. This search was last updated in February 2024. The complete review protocol is outlined in (Supplementary information1). Due to the type of samples investigated in this review (in-vitro samples), registration of this review was not possible.

**Table 1:** Databases and search keywords used in the systematic search.

Database	Keywords
Excerpta medica database (embase)	(miRNA*.mp. OR microRNA/OR microRNA*.mp. OR “micro RNA*”) AND (embryo* OR embryo/OR preimplantation embryo/OR blastocyst/OR blastocyst*.mp.) AND (Aneuploidy/OR aneuploid*.mp. OR “abnormal karyotype”.mp. or chromosome aberration OR trisomy/OR trisomy.mp. OR monosomy.mp. OR monosomy/).
MEDLARS online (Medline)	(miRNA*.mp.OR microRNA*.mp. OR MicroRNAs/OR “micro RNA*”.mp) AND (embryo*.mp. OR Blastocyst/OR “preimplantation embryo*”.mp. OR blastocyst*.mp.) AND (Aneuploidy/or aneuploid*.mp. OR “abnormal karyotype”.mp. OR Chromosome Aberrations/OR Abnormal Karyotype/OR trisomy.mp. OR Trisomy/OR monosomy.mp. OR Monosomy/)
Web of science database	(TS = miRNA* OR TS = microRNA* OR TS = “micro RNA*”) AND (TS = aneuploid* OR TS = “chromosome aberration” OR TS = “abnormal karyotype” OR TS = trisomy OR TS = Monosomy) AND (TS = embryo* OR TS = “preimplantation embryo*” OR TS = blastocyst*).
Cochrane clinical trials database	((Mesh: [miRNAs] OR miRNA* OR microRNA* OR “micro RNA”) AND (Mesh: [Aneuploidy] OR “Aneuploid” OR “abnormal karyotype” OR “chromosome* aberration” OR “Trisomy OR “Monosomy”) AND) Mesh: [Embryonic structure] OR “embryo*” OR “preimplantation embryo*” OR “Blastocyst*”))
UK national research Register	(miRNA) AND (Aneuploidy) AND (Blastocyst OR embryo)
British library (EThOS)	(miRNA) AND (Aneuploidy) AND (Blastocyst OR embryo)

### 2.2 Study selection

**Figure 1:**
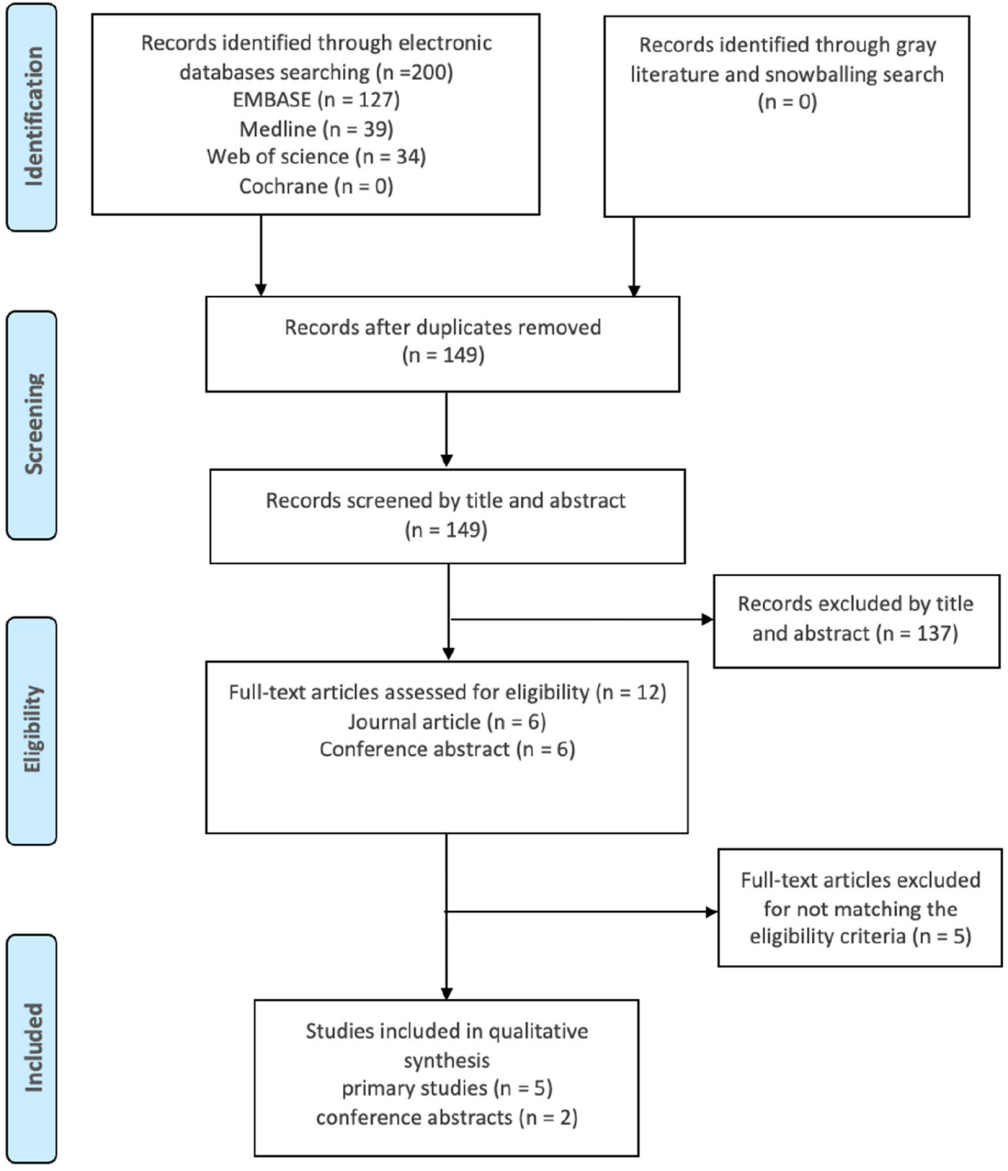
PRISMA flow diagram illustrating the flow of the search method. Records from all databases were joined then duplicates were identified and removed. The research papers were scanned by title and abstract to select the related studies. Eligible studies were elected after full-text assessment.

Study selection was performed in adherence with the Preferred Reporting Items for Systematic Reviews and Meta-Analyses (PRISMA) statement and protocols (19, 20). Search results from all databases were imported into EndNote (21), to identify and remove duplicates. The remaining records were screened based on title and abstract, and relevant studies proceeded to full-text screening and eligibility assessment (Figure 1).

### 2.3 Eligibility

The inclusion criteria for this review prioritized observational case control studies due to their suitability for investigating the relationship between miRNA and aneuploidy (Table 2). Blastocyst stage embryos were selected as the population of interest, since testing for aneuploidy is mostly performed on blastocysts, and to avoid the confounding of normal variation in miRNA profile at different embryonic stages. The main focus was on miRNAs originating from blastocysts, whether extracted directly from the blastocysts or isolated from the blastocoel fluid, trophectoderm biopsied cells, or the culture media. The studies excluded, along with reasons for their ineligibility, are listed in (Supplementary information1).

**Table 2:** Inclusion criteria of the systematic review.

Publication type	In primary literature: peer-reviewed journal articles. In grey literature: conference abstracts with informative results, theses and dissertations
Year of publication	No limit
Language	English
Study design	Case control observational studies
Population	Blastocysts
Outcome	Differentially expressed miRNAs of blastocyst origin (extracted from whole blastocysts or from trophectoderm (TE) biopsy or found in blastocoel or secreted into the culture media)

### 2.4 Quality assessment

Two reviewers (A.A. and X.G.) independently assessed the risk of bias in the reviewed papers using NHLBI-NIH assessment tools, which examine potential biases and their sources (22). The assessment was conducted using the form designed for case control studies, which includes 12 criteria. Each one was marked as either “yes” (1 point) or “no/not applicable” (0 points). The studies were rated as poor with 0–3 points, fair 4–7 points, and good 8–12 points. Any discrepancies in the assessment were resolved by the third reviewer (S.S.).

### 2.5 Data extraction and analysis

The data extracted were categorized into three sections: publication information, study descriptive characteristics, and the statistical significances of the differentially expressed miRNAs (see Tables 3, 4). Pathway annotation analysis of the commonly altered genes was conducted using Reactome and KEGG databases via miRPathDB v2.0 platforms (23).

**Table 3:** Publication information and study characteristics of the included studies.

Study title	Design	Publication type	Sample type	Maternal age (years)	Fertilization procedure	Sample size (euploid/aneuploid)	Aneuploidy testing sample/method	MiRNA measurement method/normalization	References
MicroRNA expression in the human blastocyst.	Observational case control	Peer reviewed article	Blastocysts	NA^a^	ICSI^b^	14 (9/5)	TE biopsy/aCGH^c^	Array-based real-time qPCR/U6	Rosenbluth et al. (11)
Human blastocysts exhibit unique microRNA profiles in relation to maternal age and chromosome constitution.			Blastocysts	40–44	ICSI	10 (5/5)	TE biopsy/qPCR	Array-based real-time qPCR/U6	McCallie et al. (13)
Human embryos secrete microRNAs into culture media - a potential biomarker for implantation.			Spent culture media from blastocysts	NA	ICSI	28 (19/9)	TE biopsy/aCGH	Array-based real-time qPCR/U6	Rosenbluth et al. (12)
Chaotic human blastocysts display altered microRNA regulation promoting downstream apoptotic gene transcription leading to embryo arrest.		Conference abstract	Blastocysts	Young (<35)	NA	12	NA	Array-based real-time qPCR/NA	McCallie et al. (14)
				Aged (≥40)					
Embryonic quality assessment from microRNA profiling.			Blastocysts	NA	ICSI	122	NA	miRNA sequencing/DESeq2	Almutlaq et al. (17)
microRNAs in the blastocoel fluid as accessible indicators of chromosomal normality.		Peer reviewed article	Blastocele fluid	25–28	NA	25 (8/17)	NA	real-time qPCR/U6	Esmaeilivand et al. (16)
Association of trophectoderm mRNAs and MicroRNAs with chromosomal aneuploidy of embryo.			Trophectoderm cells	NA	NA	25 (8/17)	FISH^d^	real-time qPCR/U6	Esmaeilivand et al. (18)

^a^ Data was not available.

^b^ Intra-Cytoplasmic Sperm Injection.

^c^ Array comparative genomic hybridization.

^d^ Fluorescence *in situ* hybridization.

**Table 4:** Primary outcomes of the included studies.

References	Primary outcome		
	Total number of investigated miRNAs	Number of differentially expressed miRNAs/total number of miRNAs investigated	Method used for differential expression analysis
Rosenbluth et al. (11)	754	39/754	2^−DDCt^ for fold change
McCallie et al. (13)	377	38/377	Fold change using REST© analysis software (Qiagen)
Rosenbluth et al. (12)	3	1/3	2^−DDCt^ for fold change
McCallie et al. (14)	377	13/377	Fold change using REST© analysis software (Qiagen)
Almutlaq et al. (17)	All expressed in human blastocysts	9	DESeq2 via GeneGlobe (QIAGEN)
Esmaeilivand et al. (16)	10	2/10	2^−DDCt^ for fold change
Esmaeilivand et al. (18)	10	2/10	2^−DDCt^ for fold change

## 3 Results

### 3.1 Search results

A comprehensive search of the literature identified a total of 200 publications. After the screening process, only seven studies met the inclusion criteria and were included in this review. These studies were assessed for quality and received fair to good scores. According to the Cochrane database search results, no previous reviews had been conducted on the same subject.

### 3.2 Characteristics of included studies

The initial investigations into the examined relationship were reported between 2013 and 2015, with the subsequent studies undertaken in more recent years. These studies were orchestrated by four distinct research groups, exploring miRNA profiles within blastocysts, with a specific focus on their association with numerical chromosomal abnormalities. Uniformly, array-based quantitative PCR was employed for miRNA expression in most studies, making the results more comparable, except for Almutlaq et al, who recently employed next-generation sequencing for a more comprehensive exploration of miRNAs.

The majority of the studies were performed on a limited sample size, with each study group (euploid/aneuploid) comprising no fewer than three samples. All the examined miRNAs originated from blastocysts but were derived from different locations, including whole blastocysts, blastocoel fluid, isolated trophectoderm cells, and culture media. Further study details are summarized in (Tables 3, 4).

### 3.3 Differentially expressed miRNAs

The alterations in miRNA expression profile in association with aneuploidy in blastocysts was initially reported by Rosenbluth et al. in 2013 (11), and subsequently confirmed by McCallie et al. in 2014 (13). The latter focused on a specific group of embryos derived from women aged 40–44 years. In these studies, a total of 39 and 38 differentially expressed miRNAs in aneuploid blastocysts were identified, respectively (Table 4). Among them, miR-19b, miR-517c, miR-518e and miR-522 were consistently downregulated in aneuploid blastocysts across both analyses (Table 5).

**Table 5:** Altered miRNAs with matching results across the reviewed studies.

miRNA	Other names	Chromosome location	miRNA-cluster	Publication
miR-19b	miR-19b-3p	Chr 13, Chr X	miR-17/92	Rosenbluth et al. (11)
			miR-106a-363	McCallie et al. (13)
miR-517c	miR-517c-3p	Chr 19	C19MC	Rosenbluth et al. (11)
				McCallie et al. (13)
miR-518e	miR-518e-3p	Chr 19	C19MC	Rosenbluth et al. (11)
				McCallie et al. (13)
miR-522	miR-522-3p	Chr 19	C19MC	Rosenbluth et al. (11)
				McCallie et al. (13)
miR-92a	miR-92a-3p	Chr 13, Chr X	miR-17/92	Rosenbluth et al. (11)
			miR-106a-363	McCallie et al. (14)
miR-106a	miR-106a-5p	Chr X	miR-106a-363	Rosenbluth et al. (11)
				McCallie et al. (14)
miR-30c	miR-30c-5p	Chr 1, Chr 6	miR-30 family	McCallie et al. (13)
				Esmaeilivand et al. (18)

Rosenbluth's group continued to explore miRNAs in blastocysts by analysing those that diffused into the spent culture media. They first identified blastocyst-derived miRNAs by comparing blastocyst-exposed media to control media, which showed two miRNAs, miR-372 and miR-191, of embryonic origin. Thereafter, differences in miRNA expression between euploid and aneuploid blastocysts' media were investigated, which revealed a high abundance of miR-191 in the last group (12).

In another investigation conducted by McCallie et al., the miRNA expression in blastocysts with chaotic aneuploidies, from women of different age groups, was explored (14). Thirteen miRNAs showed a significant reduction in the advanced maternal age group. Among them, only miR-92a and miR-106a were consistently downregulated in chaotic aneuploid samples, regardless of age. Both miRNAs showed similar findings in the study by Rosenbluth et al. (11).

In a previous investigation, our group aimed to establish the connection between miRNA expression and embryo quality by using next generation sequencing. The miRNA profile in human blastocysts was investigated concerning three quality factors: day of blastocyst formation, morphology and aneuploidy status. Four frequently dysregulated miRNAs, including hsa-let-7c-5p, hsa-miR-206, hsa-miR-184 and hsa-miR-203a-3p, were consistently upregulated in blastocysts with unfavourable quality, specifically in aneuploid samples (17). Discrepancies in the miRNA expression approaches across studies might account for the absence of significant alterations of these miRNAs in the other included studies, which can be attributed to the sensitivity of sequencing in identifying a broader range of miRNAs.

In a recent study, Esmaeilivand et al. explored the expression levels of predefined miRNAs associated with aneuploidy and implantation failure, including miR-345, miR-339, miR-141, miR-27b, miR-191, miR-20a, miR-30c, miR-661, miR-372, and miR-142 (16). Assessing blastocoel samples obtained from euploid and aneuploid blastocysts, the results revealed a significant increase of only miR-20a and miR-661 in the blastocoel fluid obtained from aneuploid embryos. Furthermore, the investigation extended to the examination of the selected miRNAs within the biopsied trophectoderm cells of the same blastocysts (18). The findings showed a significant downregulation of hsa-miR-30c and hsa-miR-372 levels in the aneuploid samples compared to the euploid blastocysts. Notably, the downregulation of hsa-miR-30c in the presence of chromosomal abnormalities aligns with prior findings reported by McCallie et al. (13).

Collectively, a total of seventy-one differentially expressed miRNAs were identified in the reviewed studies (Supplementary information2). Of them, only miR-19b, miR-517c, miR-518e, miR-522, miR-92a, miR-106a and miR-30c exhibited consistent results in the included studies, all demonstrating decreased levels in aneuploid blastocysts (Table 4).

### 3.4 microRNA clusters

Notably, most of the identified miRNAs are members of well-known pregnancy-associated miRNA clusters (listed in Table 5). For instance, miR-517c, miR-518e and miR-522 are encoded on C19MC, the largest human miRNA cluster. This cluster is predominantly expressed in the placenta and embryonic stem cells under normal conditions, but it was also found in certain tumours (24). Genes encoded in C19MC are maternally imprinted, with only paternal alleles naturally expressed (25). Other clusters of interest are the paralogous miR-17/92 and miR-106a-363, both located on human chromosome 13 and chromosome X and encoded miR-19b and miR-92a. The miR-106a is also a member of miR-106a-363 cluster, but it is only present on the X chromosome. These miRNA clusters are considered oncogenic to their contribution to tumorigenesis. However, they are highly expressed in embryonic stem cells, where they regulate critical events during early development such as trophoblast differentiation, gastrulation, and embryo implantation (26–28).

### 3.5 Pregnancy complications and biological function

The pathological role of the identified miRNAs has been investigated and demonstrated in various pregnancy-related problems. Changes in miR-517c levels were observed in cases of spontaneous abortions and certain placental disorders (29–34). Similarly, alterations in miR-522 and miR-518e levels showed association with implantation failures and pre-eclampsia (30, 35–37). While members of the C19MC cluster are involved in embryonic development, genes from the other clusters appear to be more engaged, or possibly more investigated, in gametes and reproductive organs. For example, knocking down the miR-17/92 cluster in mice resulted in impaired spermatogenesis, and a significant reduction of the miR-19b was observed in the semen of infertile men (38–40). These findings suggest a possible contribution of these genes to male fertility. In addition, increased expression of miR-106a has been noted in endometrial exosomes and macrovesicles (41). This gene may play a role in indicating the likelihood of implantation due to its involvement in the regulation of the embryo-endometrium dialogue (8, 41). While elevated levels of miR-30c was associated with slow cleaving of bovine embryos and poor human embryos morphokinetic, decreased expression of this miRNA was observed in the culture media from euploid un-implanted blastocysts compared to those that achieved successful implantation, indicating its contribution to the embryo competence (42–44).

### 3.6 Pathway analysis of the dysregulated miRNAs

**Figure 2:**
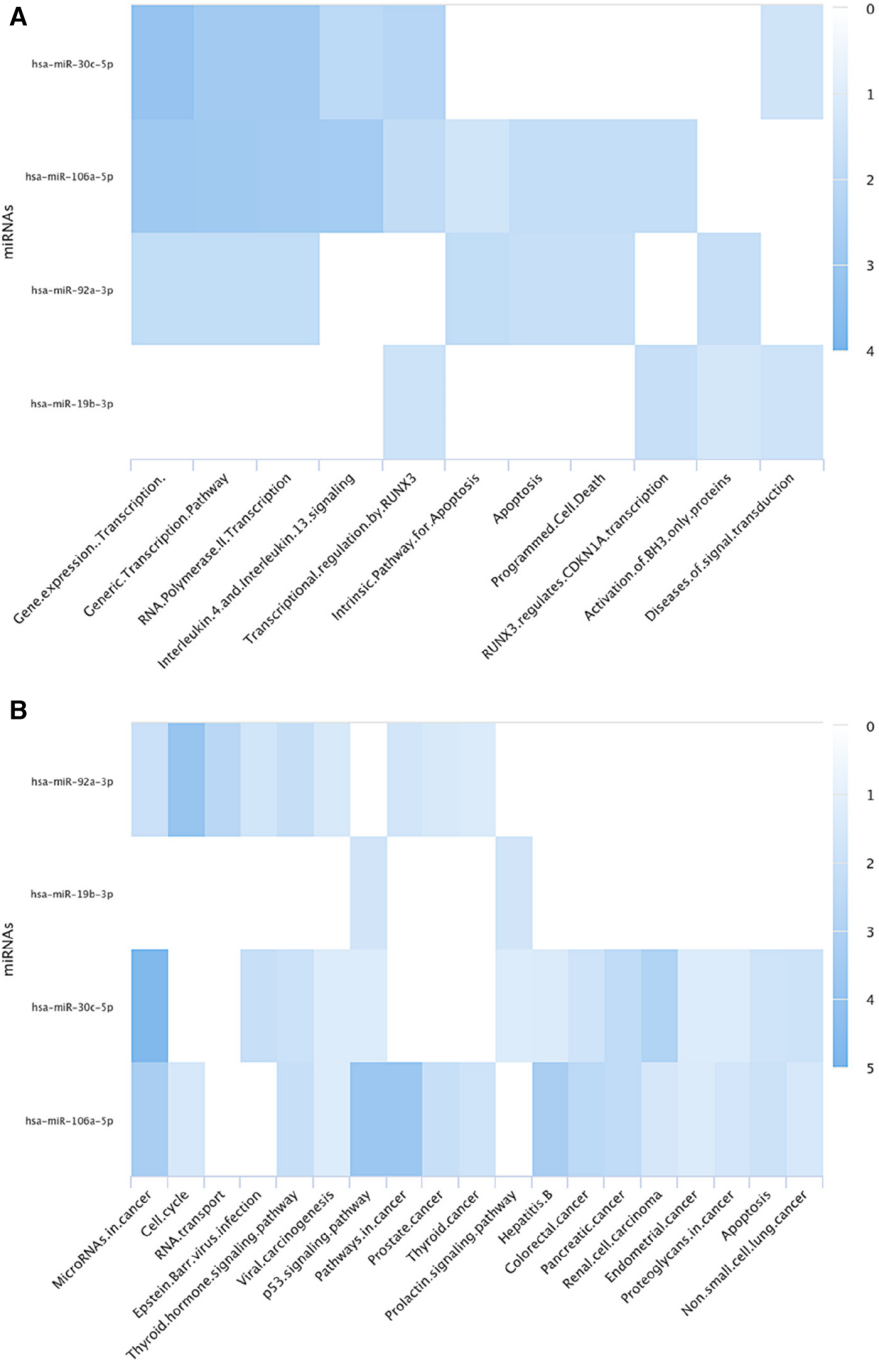
The figure illustrates pathway annotation heat maps of the identified differentially expressed miRNAs in aneuploid blastocysts using (**A**) Reactome (**B**) KEGG databases via miRPathDB 2.0. The analysis reveals that these miRNAs play a role in regulating transcription, including transcription of cell cycle genes, in apoptosis, and their involvement in various types of cancers.

To identify the potentially involved biological pathways of the identified miRNAs in this review, a pathway annotation analysis was conducted using miRPathDB 2.0 (23). This analysis, which was restricted to strong evidence studies, revealed that miR-19b, miR-92a and miR-106a were extensively investigated, regulating key cellular processes (Figure 2). The pathway analysis also revealed that the aberrant miRNAs are involved in regulating the initial phase of transcription activity, specifically the transcription by RNA polymerase II, which suggest a disturbed gene expression profile in aneuploid samples. The investigated miRNAs have also shown an influence on cell death processes, particularly apoptosis (45–47). Previous investigations revealed that inhibiting miR-92a-3p and silencing miR-106a-5p induce apoptosis in various types of cancers (48–50). Moreover, decreased levels of miR-19b was associated with promoting cell arrest and apoptosis through upregulation of the runtrelated transcription factor 3 (*RUNX3*) (51). RUNX3 also induces the expression of cyclin-dependent kinase inhibitor 1A (*CDKN1A* or P21) which promotes cell cycle arrest when elevated (52). According to the computational analysis, this pathway may also be dysregulated in aneuploid blastocysts.

Interestingly, decreased levels of tumorigenic miRNAs in aneuploid blastocysts contrast with the upregulation of these miRNAs commonly observed in cancerous cells, which frequently exhibit aneuploidies (53). This distinction may indicate that embryos have the potential to recognize the chromosomal abnormalities and respond to them. Such a response could signify a protective mechanism to prevent abnormal cell growth and ensure that only embryos with a normal chromosomal complement advance further in development.

### 3.7 Potential biomarkers

Increased research focuses on exploring embryo secretions, particularly small RNAs, to propose non-invasive markers for the embryo developmental competence. Notably, the presence of miRNAs with embryonic origin in the culture media has been observed, with a suggested association to the embryo quality and pregnancy outcomes. Varying levels of certain miRNAs, particularly miR-19b-3p, in the media have been linked to embryonic morphology (54). Additionally, other miRNAs, such as miR-92a-3p and miR-191-5p, have been proposed to potentially influences implantation success (5). In alignment with these observations on miRNA secretion into the media, some miRNAs were found in the blastocoel fluid, and their expression was related to the aneuploidy status of the embryo (16). These observations confirm the diffusing of miRNAs from embryonic cells to the extracellular fluid, which highlights the potential utilization of miRNAs as biomarkers in reproductive medicine.

## 4 Discussion

The present review summarized the association between miRNA expression and aneuploidy in blastocysts. A small number of studies have investigated this relationship, all designed to provide preliminary results. Although promising findings were obtained, the subject has been largely neglected for a significant period, with little attention devoted to further exploration. One potential explanation for this delay lies in the multifaceted relationship between miRNA and chromosomal abnormalities, compounded with the complexity of investigating aneuploidy as a singular factor due to its diverse types and levels.

Overall, the results confirm an altered miRNA expression profile in aneuploid blastocysts. Despite the small sample size and differences in the extraction site of blastocysts among the included studies, some similarities in the differentially expressed miRNAs were noticed, indicating the predictive value of the miRNAs as biomarkers. Accumulating evidence reveals the contribution of these miRNAs to critical biological pathways and their influence on embryonic development. Some were previously linked to pregnancy outcomes and complications, such as preeclampsia, while others were reported to be essential for reproductive organ functions, normal gamete production and implantation (5, 24, 25, 31, 32, 35, 38–40). Furthermore, some were found to be associated with embryo development and morphology, suggesting that these miRNAs can depict the overall quality of developing embryo and, therefore, could predict likelihood of implantation (43, 54).

These miRNAs generally fall into two categories: tumorigenic and pregnancy-related miRNAs. Notably, all the involved miRNA clusters are typically expressed in embryos but are also found in some cancers, which highlight the similarity between early embryos and cancerous cells as both are undifferentiated cells that commonly develop chromosomal abnormalities. Interestingly, the miRNAs that are known to promote proliferation in cancer were downregulated in aneuploid embryos, suggesting a distinct pattern of gene expression regulation in aneuploid blastocysts compared to cancer cells (53). Interestingly, the miRNAs that are known to promote proliferation in cancer were downregulated in aneuploid embryos, suggesting a distinct pattern of gene expression regulation in aneuploid blastocysts compared to cancer cells.

Moreover, the pathway annotation revealed that certain types of cancers, particularly the reproductive-related ones, like prostate, endometrial and thyroid cancers, are significantly associated with the differentially expressed miRNAs in aneuploid blastocysts. Ultimately, gaining an enhanced understanding of the relationship between miRNAs and aneuploidy has the potential to advance not only the assessment of embryo quality but also the understanding of the molecular pathology of various conditions characterized by chromosomal abnormalities and dysregulated miRNAs.

Additionally, the consistent dysregulation of miRNAs encoded on Chromosomes 13, 19 and X in aneuploid blastocysts provide insights into the highly involved chromosomes and, thus, their encoded genes, when the chromosomal complement is disturbed. Furthermore, the fact that some of the altered miRNAs in the presence of chromosomal abnormalities have paternal origin highlight the critical role of paternal genome in regulating early developmental events, as previously suggested (55). Indeed, further investigations into these observations would enhance our understanding on the embryo cellular mechanisms responding to chromosomal abnormalities in early development.

Recent research showed an increased interest in embryo secretions as potential biomarkers, particularly small RNAs. Several studies have successfully detected miRNAs derived from blastocysts and correlated them with embryo quality or pregnancy outcome (4, 5). Notably, some of the identified miRNAs associated with the aneuploidy status of blastocysts were found in the culture media (12, 54). The utilisation of miRNAs as quality biomarkers for preimplantation embryos offers a multitude of advantages, notably the non-invasive extraction from culture media (4). This approach holds the promise of reducing the potential harm caused by TE biopsy and may provide a more comprehensive picture of the genetic status of the embryo. In compression to the current method of chromosomal testing for aneuploidy, utilising gene expression to predict the embryo competence, including the aneuploidy status, could address the challenge of technical errors leading to false aneuploidy calls in the PGT-A. Nevertheless, further studies and accumulation of substantial data are needed to establish and solidify this approach.

## 5 Conclusion

The present review conducted a literature search to explore the association between miRNA expression and aneuploidy in blastocysts. Overall, the findings revealed that the miRNA profile was frequently altered in aneuploid embryos, confirming the inspected association. However, additional investigations on a larger sample size, including different types of aneuploidies, and minimizing biological variations are required to gain deeper insights into this relationship.

## Data Availability

The original contributions presented in the study are included in the article/Supplementary Material, further inquiries can be directed to the corresponding author.
