# Supplementary material for: Differentially expressed microRNAs in aneuploid preimplantation blastocysts: a systematic review
Source: Front Reprod Health. 2024 Mar 14;6:1370341. doi: 10.3389/frph.2024.1370341 (PMC10973143; doi:10.3389/frph.2024.1370341)
Supplement: Supplementary file 1 [file Datasheet1.docx]

**Appendix A**

**Supplementary information1**

Study design:
Systematic review

Title:
Differentially expressed microRNAs in aneuploid preimplantation blastocysts: a systematic review

Research question:
Does the expression profile of miRNA change in aneuploid preimplantation blastocysts when compared to euploid ones?

Aim:
MiRNA expression is a promising molecular tool for diagnosis and prognosis of certain diseases. The potential of utilising miRNA markers for embryo quality assessment in preimplantation genetic testing (PGT) necessitates finding a relationship between miRNA expression and aneuploidy, the primary indicator in embryo transfer selection. This review aims to explore the literature and integrate the existing information on the relationship between aneuploidy and miRNA expression in blastocysts.

Defining the question using PICO:
**Population:** Pre-implantation embryos (blastocysts).
**Intervention (exposure):** aneuploidy.
**Comparison (control):** euploid blastocysts.
**Outcome:** miRNA differential expression analysis results.

.

Table A-1: Inclusion criteria

| Publication type | In primary literature: peer-reviewed journal articles. In grey literature: conference abstracts with informative results, theses and dissertations. |
| --- | --- |
| Year of publication | No limit. |
| Language | English. |
| Study design | Case control observational studies. |
| Population | Blastocyst embryos. |
| Outcome | Differentially expressed miRNAs of blastocyst origin (extracted from whole blastocysts or from trophectoderm (TE) biopsy or found in blastocoel or secreted into the culture media.) |

Electronic databases to use:
Excerpta Medica database (Embase)
MEDLARS Online (Medline)
Web of Science database
Cochrane clinical trials database

Internet sources to search:
U.K. National Research Register
British Library (EThOS)

Type of literature included:
Peer reviewed journal articles
Reference lists of eligible studies
Conference abstracts
Theses and dissertations

Table A-2: Search strategy using Boolean operators

| Database | Keywords |
| --- | --- |
| Excerpta Medica database (Embase) | (miRNA*.mp. OR microRNA/ OR microRNA*.mp. OR “micro RNA*”) AND (embryo* OR embryo/ OR preimplantation embryo/ OR blastocyst/ OR blastocyst*.mp.) AND (Aneuploidy/ OR aneuploid*.mp. OR "abnormal karyotype".mp. or chromosome aberration OR trisomy/ OR trisomy.mp. OR monosomy.mp. OR monosomy/). |
| MEDLARS Online (Medline) | (miRNA*.mp.OR microRNA*.mp. OR MicroRNAs/ OR "micro RNA*".mp) AND (embryo*.mp. OR Blastocyst/ OR "preimplantation embryo*".mp. OR blastocyst*.mp.) AND (Aneuploidy/ or aneuploid*.mp. OR "abnormal karyotype".mp. OR Chromosome Aberrations/ OR Abnormal Karyotype/ OR trisomy.mp. OR Trisomy/ OR monosomy.mp. OR Monosomy/) |
| Web of Science database | (TS=miRNA* OR TS= microRNA* OR TS= "micro RNA*") AND (TS= aneuploid* OR TS= "chromosome aberration" OR TS="abnormal karyotype" OR TS= trisomy OR TS= Monosomy) AND (TS= embryo* OR TS= "preimplantation embryo*" OR TS= blastocyst*). |
| Cochrane clinical trials database | ((Mesh: [miRNAs] OR miRNA* OR microRNA* OR “micro RNA”) AND (Mesh: [Aneuploidy] OR “Aneuploid” OR “abnormal karyotype” OR “chromosome* aberration” OR ‘Trisomy OR “Monosomy”) AND) Mesh: [Embryonic structure] OR “embryo*” OR “preimplantation embryo*” OR “Blastocyst*)) |
| UK National Research Register | (miRNA) AND (Aneuploidy) AND (Blastocyst OR embryo) |
| British Library (EThOS) | (miRNA) AND (Aneuploidy) AND (Blastocyst OR embryo) |

Table A-3: Excluded studies

| Excluded study for eligibility | Publication type | Justification |
| --- | --- | --- |
| Differential expression of micro-RNA in day 5 human euploid and aneuploid blastocysts. | Abstract | Repeated. Results were reported in an included study (MicroRNA expression in the human blastocyst). |
| Human blastocysts exhibit unique microrna profiles in relation to maternal age and chromosome constitution. | Abstract | Repeated. Results were reported in an included study (Human blastocysts exhibit unique microrna profiles in relation to maternal age and chromosome constitution). |
| MicroRNA in culture media from human blastocysts exhibits a distinct signature that correlates with embryonic chromosomes and IVF outcome. | Abstract | Repeated. Results were reported in an included study (Differential expression of micro-RNA in day 5 human euploid and aneuploid blastocysts). |
| MicroRNA testing: A novel, non-invasive technique to detect aneuploidy and live birth potential in human embryos. | Abstract | Repeated. Results were reported in an included study (Differential expression of micro-RNA in day 5 human euploid and aneuploid blastocysts). |
| Correlation between differential expression of microRNA and quality of embryos | Article | Language - Chinese |
