# Supplementary material for: Differentially expressed microRNAs in aneuploid preimplantation blastocysts: a systematic review
Source: Front Reprod Health. 2024 Mar 14;6:1370341. doi: 10.3389/frph.2024.1370341 (PMC10973143; doi:10.3389/frph.2024.1370341)
Supplement: Supplementary file 2 [file Datasheet2.docx]

**Appendix B**

**Supplementary information 2**

Table A-3: Repeatedly altered miRNAs in aneuploid versus euploid embryos.

| Study | miRNA | Regulation | Matching |
| --- | --- | --- | --- |
| Rosenbluth et al., 2013 / McCallie et al, 2015 | miR-106a | Down/Downregulated | Matched |
| Rosenbluth et al., 2013 / McCallie et al., 2014 | miR-146b-5p | Down/Up regulated | Not matched |
| Rosenbluth et al., 2013 / McCallie et al., 2014 | miR-19b | Down/Downregulated | Matched |
| Rosenbluth et al., 2013 / McCallie et al., 2014 | miR-200c | Down/Up regulated | Not matched |
| Rosenbluth et al., 2013 / McCallie et al., 2014 | miR-320 | Down/Up regulated | Not matched |
| Rosenbluth et al., 2013 / McCallie et al., 2014 | miR-367 | Down/Up regulated | Not matched |
| Rosenbluth et al., 2013 / McCallie et al., 2014 | miR-371-3p | Down/Up regulated | Not matched |
| Rosenbluth et al., 2013 / McCallie et al., 2014 /  Esmaeilivand et al, 2023 | miR-372 | Down/Up/Downregulated | Not matched |
| Rosenbluth et al., 2013 / McCallie et al., 2014 | miR-373 | Down/Up regulated | Not matched |
| Rosenbluth et al., 2013 / McCallie et al., 2014 | miR-517c | Down/Downregulated | Matched |
| Rosenbluth et al., 2013 / McCallie et al., 2014 | miR-518e | Down/Downregulated | Matched |
| Rosenbluth et al., 2013 / McCallie et al., 2014 | miR-522 | Down/Downregulated | Matched |
| Rosenbluth et al., 2013 / McCallie et al., 2014 | miR-93 | Down/Up regulated | Not matched |
| Rosenbluth et al., 2013 / McCallie et al., 2015 | miR-92a | Down/Downregulated | Matched |
| Rosenbluth et al., 2013 / Esmaeilivand et al, 2022 | miR-20a | Down/Up regulated | Not matched |
| McCallie et al., 2014 /  Esmaeilivand et al, 2023 | miR-30c | Up/Down regulated | Not matched |

Table A-4: Statistically significant differentially expressed miRNAs in euploid versus aneuploid embryos.

| Reference | differentially expressed miRNAs | Regulation in aneuploid to euploid | Fold change as reported | p-value |
| --- | --- | --- | --- | --- |
| Rosenbluth et al., 2013 | hsa-miR-106a  hsa-miR-1276  hsa-miR-141  hsa-miR-146b-5p  hsa-miR-148a  hsa-miR-155  hsa-miR-17  hsa-miR-19a  hsa-miR-19b  hsa-miR-200c  hsa-miR-20a  hsa-miR-26b#  hsa-miR-27b  hsa-miR-30b  hsa-miR-320  hsa-miR-339-3p  hsa-miR-345  hsa-miR-34b  hsa-miR-367  hsa-miR-371-3p  hsa-miR-372  hsa-miR-373  hsa-miR-380-5p  hsa-miR-487b  hsa-miR-509-5p  hsa-miR-517c  hsa-miR-518a-3p  hsa-miR-518c  hsa-miR-518e  hsa-miR-519a  hsa-miR-522  hsa-miR-566  hsa-miR-590-3p  hsa-miR-597  hsa-miR-645  hsa-miR-660  hsa-miR-886-3p  hsa-miR-92a  hsa-miR-93 | Downregulated  Downregulated  Downregulated  Downregulated  Downregulated  Downregulated  Downregulated  Downregulated  Downregulated  Downregulated  Downregulated  Downregulated  Downregulated  Downregulated  Downregulated  Downregulated  Downregulated  Downregulated  Downregulated  Downregulated  Downregulated  Downregulated  Downregulated  Up regulated  Downregulated  Downregulated  Downregulated  Downregulated  Downregulated  Downregulated  Downregulated  Downregulated  Downregulated  Downregulated  Downregulated  Downregulated  Downregulated  Downregulated  Downregulated | 1.15  6.22  5.49  0.61  4.19  4.01  1.38  1.40  0.94  1.08  1.78  3.07  4.29  1.22  0.48  3.69  1.69  5.26  0.90  0.88  0.67  1.52  3.17  −3.35  1.33  1.28  3.79  2.81  1.63  1.15  2.15  4.08  3.45  4.82  5.00  2.69  0.64  1.73  3.75 | P= <0.05  P= <0.05  P= <0.05  P= <0.05  P= <0.05  P= <0.05  P= <0.05  P= <0.05  P= <0.05  P= <0.05  P= <0.05  P= <0.05  P= <0.05  P= <0.05  P= <0.05  P= <0.05  P= <0.05  P= <0.05  P= <0.05  P= <0.05  P= <0.05  P= <0.05  P= <0.05  P= <0.05  P= <0.05  P= <0.05  P= <0.05  P= <0.05  P= <0.05  P= <0.05  P= <0.05  P= <0.05  P= <0.05  P= <0.05  P= <0.05  P= <0.05  P= <0.05  P= <0.05  P= <0.05 |
| McCallie et al., 2014 | hsa-miR-106b-5p  hsa-miR-125a-5p  hsa-miR-146b-5p  hsa-miR-193b  hsa-miR-195-5p  hsa-miR-19b  hsa-miR-200c-3p  hsa-miR-20b-5p  hsa-miR-218-5p  hsa-miR-28-3p  hsa-miR-302a-3p  hsa-miR-302b-3p  hsa-miR-302c  hsa-miR-30c-5p  hsa-miR-320  hsa-miR-342-3p  hsa-miR-367-3p  hsa-miR-371a-3p  hsa-miR-372  hsa-miR-373  hsa-miR-374a  hsa-miR-374b-5p  hsa-miR-381-3p  hsa-miR-454-3p  hsa-miR-484  hsa-miR-508-3p  hsa-miR-515-3p  hsa-miR-517c  hsa-miR-518b  hsa-miR-518e  hsa-miR-518f-3p  hsa-miR-520b-3p  hsa-miR-520g  hsa-miR-521  hsa-miR-522  hsa-miR-628-5p  hsa-miR-886-5p  hsa-miR-93 | Up regulated  Downregulated  Up regulated  Up regulated  Up regulated  Downregulated  Up regulated  Up regulated  Up regulated  Up regulated  Up regulated  Up regulated  Downregulated  Up regulated  Up regulated  Up regulated  Up regulated  Up regulated  Up regulated  Up regulated  Up regulated  Up regulated  Up regulated  Up regulated  Up regulated  Up regulated  Up regulated  Downregulated  Downregulated  Downregulated  Up regulated  Up regulated  Downregulated  Up regulated  Downregulated  Up regulated  Downregulated  Up regulated | 2.1  <0.5  2.61  1.9  3.2  0.5  1.7  2.2  >10  1.5  3.8  2.7  0.5  2.4  1.4  1.5  1.6  1.5  1.3  2.3  1.4  1.9  >10  2.6  1.5  >10  2.0  0.5  <0.5  0.5  >10  2.2  0.4  3.4  0.7  >10  0.2  1.4 | P= <0.05  P= <0.05  P= <0.05  P= <0.05  P= <0.05  P= <0.05  P= <0.05  P= <0.05  P= <0.05  P= <0.05  P= <0.05  P= <0.05  P= <0.05  P= <0.05  P= <0.05  P= <0.05  P= <0.05  P= <0.05  P= <0.05  P= <0.05  P= <0.05  P= <0.05  P= <0.05  P= <0.05  P= <0.05  P= <0.05  P= <0.05  P= <0.05  P= <0.05  P= <0.05  P= <0.05  P= <0.05  P= <0.05  P= <0.05  P= <0.05  P= <0.05  P= <0.05  P= <0.05 |
| Rosenbluth et al., 2014 | hsa-miR-191-5p | Up regulated | 4.7 | P= 0.031 |
| McCallie et al, 2015 | hsa-miR-106a  hsa-miR-92a  other 11 miRNAs^1^ | Downregulated  Downregulated  Downregulated | N/A  N/A  N/A | P= <0.05  P= <0.05  P= <0.05 |
| Almutlaq et al, 2022 | hsa-let-7c-5p  hsa-miR-206  hsa-miR-184  hsa-miR-203a-3p | Up regulated  Up regulated  Up regulated  Up regulated | >2  >2  >2  >2 | P= <0.05  P= <0.05  P= <0.05  P= <0.05 |
| Esmaeilivand et al, 2022 | hsa-miR-661  hsa-miR-20a | Up regulated  Up regulated | N/A  N/A | P= <0.05  P= <0.05 |
| Esmaeilivand et al, 2023 | hsa-miR-30c hsa-miR-372 | Downregulated  Downregulated | N/A  N/A | P= 0.008 P= 0.01 |
